# Dynamic evolution of readmission risk factors across short-, medium-, and long-term horizons in type 2 diabetes: a machine learning-based predictive modeling study with SHAP interpretability

**DOI:** 10.3389/fendo.2026.1827506

**Published:** 2026-06-22

**Authors:** Lei Li, Sheng Jiang

**Affiliations:** Department of Endocrinology, The First Affiliated Hospital of Xinjiang Medical University, Urumqi, China

**Keywords:** artificial neural network, dynamic risk factors, machine learning, readmission prediction, SHAP, temporal validation, type 2 diabetes

## Abstract

**Background:**

T2DM readmission risk factors may evolve across time windows, but this dynamic remains poorly understood.

**Methods:**

This retrospective cohort study developed nine machine learning models to predict 30-day, 60-day, and 365-day readmission in 12,041 T2DM patients (with an additional 2,007 patients used for temporal validation of the 30-day and 60-day models). Feature selection was performed using LASSO and Boruta. SHAP analysis was used for interpretability, with temporal validation performed for short- and medium-term models.

**Results:**

ANN achieved the highest AUROC for 30-day and 60-day predictions. Random forest showed competitive performance for 365-day prediction. SHAP analysis revealed a dynamic evolution: age dominated the 30-day window; length of hospital stay and inflammatory markers (SII, SIRI) emerged as key predictors in the 60-day window; and diabetes-specific chronic complications dominated the 365-day window.

**Conclusion:**

Model selection should be time window-specific: ANN for short/medium-term, random forest for long-term prediction. Risk factors shift from acute vulnerability to inflammatory burden and then to chronic complications, supporting dynamic risk monitoring in T2DM patients.

## 1 Introduction

Type 2 diabetes mellitus (T2DM) is one of the most prevalent chronic non-communicable diseases worldwide (1–3), and its rising prevalence has imposed a heavy burden on healthcare systems (4–7). According to the 11th edition of the *IDF Diabetes Atlas* (2025) published by the International Diabetes Federation (IDF) (8), there are 589 million adults with diabetes globally, accounting for 11.1% of the adult population. More concerningly, approximately 252 million patients are unaware that they have diabetes, placing them at a higher risk of complications and premature death. The readmission rate among T2DM patients is significantly higher than that among non-diabetic individuals, reaching as high as 14%–20% within 30 days after discharge (9). This not only reflects inadequate disease management but is also directly associated with poor patient prognosis, worsening complications, and waste of healthcare resources (10–12).

The Centers for Medicare & Medicaid Services (CMS) in the United States has made the readmission rate one of the core metrics for hospital quality assessment and reimbursement, highlighting its importance (13). The root cause of readmission in T2DM patients is often not a single acute event but rather the progression of systemic disease resulting from long-term poor glycemic control (14–17). Readmission risk factors may differ significantly across time windows: in the short term, they may be related to acute metabolic disturbances, infections, or initial treatment responses; in the medium to long term, they are more influenced by multidimensional factors such as chronic complication progression, medication adherence, and social support. However, current clinical guidelines do not yet recommend specific predictive tools for different time windows (e.g., 30, 60, and 365 days), and there is a lack of systematic understanding of the dynamic evolution of risk factors (18, 19).

In recent years, machine learning (ML) methods have shown great potential in disease risk prediction due to their advantages in handling high-dimensional, nonlinear, and multicollinear data (20). Algorithms such as random forest, XGBoost, LightGBM, and artificial neural networks (ANNs) have been successfully applied to predict diabetes readmission and generally outperform traditional logistic regression models (21–23). However, these models are often regarded as “black boxes,” and their prediction mechanisms lack clinical interpretability, which limits their widespread adoption in clinical practice. To address this, the introduction of SHAP (Shapley Additive Explanations) provides global and local feature attribution capabilities for models, enabling researchers to quantify the direction and magnitude of each risk factor’s contribution to the prediction outcome.

Although studies have used machine learning to build prediction models for T2DM readmission, the vast majority still focus on short-term readmission, and few have explored the differences in risk factors for medium- and long-term readmission and their dynamic evolution. Therefore, based on a single-center, large-sample clinical cohort, this study used nine machine learning algorithms to build prediction models for 30-day, 60-day, and 365-day readmission in T2DM patients. By systematically comparing model performance across different time windows, we selected the optimal models, combined LASSO and Boruta algorithms for feature selection, and further applied SHAP to reveal differences in the contributions of key predictors and their dynamic evolutionary trends across time windows. This study aims to provide a set of time-resolved risk stratification tools for clinical practice, facilitating a shift from static risk identification to dynamic risk monitoring, thereby offering data-driven support for the development of individualized follow-up and intervention strategies.

## 2 Materials and methods

### 2.1 Study population

**Figure 1:**
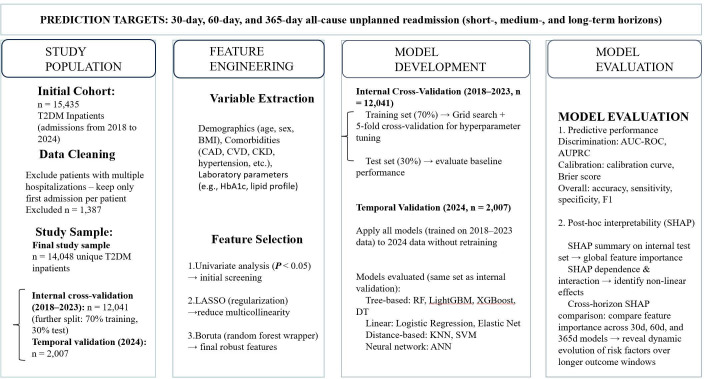
Study design for 30-day, 60-day, and 365-day readmission in T2DM patients.

This single-center retrospective cohort study was conducted at The First Affiliated Hospital of Xinjiang Medical University. A total of 14,048 patients with type 2 diabetes mellitus (T2DM) hospitalized between November 2018 and July 2024 were initially screened from the hospital’s electronic medical record system. The follow-up end date for this study was July 2024, and all readmission events occurring within 30, 60, or 365 days after discharge were captured (**Figure 1**).

Inclusion criteria were**:** (1) hospitalized patients with a primary diagnosis of T2DM; (2) availability of complete demographic and clinical data.

Exclusion criteria were: (1) patients with type 1 diabetes, gestational diabetes, or other specific types of diabetes; (2) patients transferred from or to another hospital; (3) patients who died during hospitalization; (4) patients with missing data on key outcome variables.

This retrospective study was approved by the Ethics Committee of the First Affiliated Hospital of Xinjiang Medical University (approval No. 20251218-34) and was conducted in accordance with the Declaration of Helsinki. The need for informed consent was waived by the ethics committee because the study involved only de-identified data from routine clinical care and posed no more than minimal risk to patients.

### 2.2 Study variables and definitions

#### 2.2.1 Study variables

Predictor variables were selected based on clinical relevance and literature review, including demographic characteristics (age, sex, marital status, occupation), comorbidities (hypertension, dyslipidemia, coronary artery disease, myocardial infarction, lacunar infarction, diabetic retinopathy, diabetic nephropathy, chronic kidney disease, chronic obstructive pulmonary disease, sleep disorder, osteoporosis, tumor), laboratory parameters (fasting blood glucose, glycated hemoglobin, triglycerides, total cholesterol, neutrophil count, lymphocyte count, monocyte count, platelet count), and derived inflammatory indices (SIRI, SII).

#### 2.2.2 Readmission

Readmission was defined as re-admission to the same hospital after discharge, with the primary diagnosis still being type 2 diabetes. The time to readmission was calculated as the number of days from the patient’s discharge date to the subsequent admission date. Transfers between different wards within the same hospital or transfers to other hospitals were not counted as readmissions. Based on the time window, this study classified readmissions as 30-day (within 30 days after discharge), 60-day (within 60 days), and 365-day (within 365 days). The readmission group included patients with a record of readmission between November 2018 and July 2024, after excluding duplicate hospitalization information; the non-readmission group comprised patients who were hospitalized only once during the same period.

#### 2.2.3 Variable encoding

For tree-based models (random forest, XGBoost, LightGBM, decision tree), categorical variables were encoded using integer label encoding because tree-based algorithms can handle arbitrary ordinal encoding without imposing linear order assumptions. For linear models, kernel-based models, and neural networks (logistic regression, elastic net, SVM, KNN, ANN), categorical variables were one-hot encoded to avoid imposing an artificial ordinal structure.

### 2.3 Missing data handling and preprocessing

Missing value imputation: Multiple imputation was used to handle missing values in biochemical indicators to reduce potential bias and improve statistical power. Multiple imputation by chained equations was performed using the mice package in R, incorporating all analysis variables (including outcome variables and covariates) as predictors. Predictive mean matching (PMM) was used to impute continuous missing variables, ensuring that imputed values fell within the original observed range. Categorical variables (e.g., sex, marital status, chronic disease indicators) were converted to factor type before imputation and were used as predictors for imputation but were not themselves imputed. A total of five imputed datasets were generated, with 10 iterations of the chained equations for each dataset. Convergence of the imputation process and the consistency between the distributions of imputed and observed values were checked using visual trace plots, density comparison plots, and strip plots. The results showed good convergence of the imputation model and reasonable distribution of imputed values.

Standardization: Continuous variables (age, length of hospital stay, SIRI, SII) were standardized using Z-scores. The standardizer was fitted only on the training set and then applied to the training set, internal validation set, and temporal validation set to prevent data leakage.

### 2.4 Imbalanced data handling strategies

The readmission rates in all three time windows were low (30-day: 1.86%; 60-day: 2.41%; 365-day: 6.05%), resulting in a severe class imbalance problem, particularly for the 30-day and 60-day models. For different models, the following imbalance handling strategies were implemented:

Random forest and decision tree: The class_weight=‘balanced’ parameter was used to automatically adjust weights inversely proportional to class frequencies.

XGBoost: The scale_pos_weight parameter was set to the ratio of negative to positive samples in the training set.

LightGBM: The is_unbalance=True parameter was used to automatically weight the minority class.

Logistic regression and elastic net: The class_weight=‘balanced’ parameter was used.

ANN, KNN, and SVM: These models do not natively support class weighting; therefore, we addressed class imbalance via threshold optimization: the optimal classification threshold was determined by maximizing the Youden index (sensitivity + specificity – 1) on the validation set, and PR-AUC was used as the primary evaluation metric. All reported metrics are based on the Youden-optimized threshold.

### 2.5 Data splitting and temporal validation strategy

Patients admitted between November 2018 and December 2023 (n = 12,041) were stratified and randomly split into a training set (n = 8,429) and an internal validation set (n = 3,612) at a 7:3 ratio, maintaining consistent readmission rates across all time windows in both groups. These 12,041 patients were used for model training and internal validation for the 30-day, 60-day, and 365-day readmission endpoints.

An additional 2,007 patients admitted between January 2024 and July 2024 were selected as an independent temporal validation set. Because patients admitted in 2024 could not complete a full 365 days of follow-up, this temporal validation set was used only for the 30-day and 60-day models; no temporal validation was performed for the 365-day readmission prediction. All preprocessing steps (including standardization and encoding) were fitted exclusively on the training set and then applied to the internal validation and temporal validation sets to prevent data leakage.

### 2.6 Feature selection

To avoid data leakage, all feature selection procedures were performed only on the training set. LASSO regression used 10-fold cross-validation to determine the optimal penalty parameter (λ). The Boruta algorithm was applied to the full training set with its default random forest-based significance tests, without additional cross-validation. Multicollinearity was assessed using variance inflation factor (VIF); all VIF values were <5.

### 2.7 Model development and hyperparameter optimization

Nine machine learning algorithms were developed and compared: random forest (RF), LightGBM, XGBoost, logistic regression, K-nearest neighbors (KNN), support vector machine (SVM), artificial neural network (ANN), decision tree (DT), and elastic net. All models were implemented using scikit-learn (version 1.3.0); gradient boosting models used XGBoost 2.0.1 and LightGBM 4.6.0.

Grid search combined with 5-fold cross-validation was used for hyperparameter tuning. The training set was divided into 5 folds; in each iteration, 4 folds were used for training and 1 fold for validation. After 5 iterations, the average performance across all folds was calculated. The hyperparameter configuration with the highest average predictive accuracy was selected as optimal. All models were trained using a global random seed (random_state = 1000) to ensure reproducibility.

### 2.8 Evaluation metrics

Model performance was comprehensively evaluated using discrimination, calibration, and clinical utility metrics.

Discrimination: AUROC and AUPRC were used.

Threshold-dependent metrics: Sensitivity, specificity, positive predictive value, negative predictive value, and F1 score were calculated based on the Youden-optimized threshold on the validation set.

Calibration: Brier score, calibration intercept, and calibration slope were used.

Model comparison: DeLong’s test (two-sided, α = 0.05) was used for statistical comparison of AUROC.

Interpretability: The SHAP algorithm was used to evaluate the contribution of each feature to the model output.

### 2.9 Statistical analysis

All analyses were performed using Python 3.11.5 and R 4.3.1. Continuous variables not following a normal distribution were presented as median (interquartile range) [*M* (*P*_25_, *P*_75_)] and compared between groups using non-parametric tests. Categorical variables were presented as frequencies and percentages (n, %) and compared between groups using the χ² test. A *P*-value < 0.05 was considered statistically significant.

## 3 Results

### 3.1 Participant characteristics

A total of 8,429 patients from the 2018–2023 cohort were included in the training and internal validation sets after stratified splitting (7:3 ratio) to maintain the outcome distribution. Among them, 157 patients (1.86%) experienced 30-day readmission, whereas 8,272 (98.14%) did not. Significant differences between the two groups were observed in age, length of hospital stay, SIRI, marital status, lacunar infarction, dyslipidemia, COPD, sleep disorders, and osteoporosis (all *P* < 0.05).

For 60-day readmission, 203 patients (2.41%) were readmitted, and 8,226 (97.59%) were not. Significant differences were found in age, length of stay, SIRI, SII, marital status, myocardial infarction, lacunar infarction, uremic stage of renal failure, diabetic retinopathy, dyslipidemia, COPD, sleep disorders, osteoporosis, and tumor (all *P* < 0.05).

For 365-day readmission, 510 patients (6.05%) were readmitted, and 7,919 (93.95%) were not. Significant differences between groups were observed in age, length of stay, SIRI, SII, HbA1c, marital status, occupation, coronary artery disease, myocardial infarction, lacunar infarction, CKD stage 3–5, diabetic retinopathy, dyslipidemia, COPD, sleep disorders, and osteoporosis (all *P* < 0.05) (**Tables 1**, **2**).

**Table 1:** Readmission counts and rates in the 2018–2023 cohort (training and internal validation sets).

Time window	Total (readmission rate)	Training set (readmission rate)	Validation set (readmission rate)
30d	224/12041(1.86%)	157/8429(1.86%)	67/3612(1.85%)
60d	290/12041(2.41%)	203/8429(2.41%)	87/3612(2.41%)
365d	728/12041(6.05%)	510/8429(6.05%)	218/3612(6.04%)

**Table 2:** Baseline characteristics of non-readmission and readmission groups.

Variables	30-day non-readmission group (n=8,272)	30-day readmission group (n=157)	Z/χ²	P	60-day non-readmission group (n=8,226)	60-day readmission group (n=203)	Z/χ²	P	365-day non-readmission group (n=7,919)	365-day readmission group (n=510)	Z/χ²	P
Age, M (Q_1_, Q_3_)	58.00(50.00,66.00)	61.00(53.00,72.00)	Z=-3.83	<0.001	58.00(50.00,66.00)	60.00(52.00,72.00)	Z=-3.67	<0.001	58.00(50.00,66.00)	61.00(52.00,68.00)	Z=-4.57	<0.001
Length of hospital stay, M (Q_1_, Q_3_)	6.00(5.00,7.00)	5.00(3.00,7.00)	Z=-5.81	<0.001	6.00(5.00,7.00)	5.00(2.50,7.00)	Z=-6.86	<0.001	6.00(5.00,7.00)	6.00(4.00,7.00)	Z=-6.21	<0.001
SIRI, M (Q_1_, Q_3_)	0.91(0.62,1.40)	1.06(0.65,1.65)	Z=-2.08	0.037	0.91(0.62,1.39)	1.06(0.68,1.60)	Z=-2.66	0.008	0.92(0.63,1.40)	0.98(0.67,1.42)	Z=-2.35	0.019
SII, M (Q_1_, Q_3_)	400.27(280.34,592.56)	452.42(299.50,636.22)	Z=-1.71	0.087	400.78(279.08,591.87)	452.42(294.68,679.70)	Z=-2.14	0.033	399.26(280.34,595.50)	435.94(311.04,640.40)	Z=-3.33	<0.001
HbA1c, M (Q_1_, Q_3_)	8.40(7.06,10.20)	8.30(6.90,9.98)	Z=-1.00	0.318	8.35(7.00,10.11)	8.30(6.90,9.73)	Z=-1.30	0.195	8.40(7.00,10.20)	8.16(6.80,9.84)	Z=-2.74	0.006
TyG-BMI, M (Q_1_, Q_3_)	236.52(210.30,266.17)	234.97(214.33,258.14)	Z=-0.43	0.667	235.69(209.51,265.26)	234.97(214.00,259.05)	Z=-0.15	0.88	236.84(210.47,265.92)	233.08(210.25,259.69)	Z=-1.76	0.078
Sex, n (%)			χ²=0.02	0.89			χ²=0.05	0.83			χ²=0.01	0.924
Male	5313(64.23)	100(63.69)			5248(63.80)	131(64.53)			5032(63.54)	323(63.33)		
Female	2959(35.77)	57(36.31)			2978(36.20)	72(35.47)			2887(36.46)	187(36.67)		
Marital status, n (%)			–	0.011			–	0.003			χ²=12.23	0.007
Married	7507(90.75)	132(84.08)			7505(91.24)	172(84.73)			7204(90.97)	450(88.24)		
Widowed	418(5.05)	13(8.28)			397(4.83)	17(8.37)			401(5.06)	33(6.47)		
Divorced	130(1.57)	7(4.46)			127(1.54)	9(4.43)			121(1.53)	17(3.33)		
Unmarried	217(2.62)	5(3.18)			197(2.39)	5(2.46)			193(2.44)	10(1.96)		
Occupation, n (%)			χ²=6.94	0.326			χ²=5.37	0.498			χ²=20.03	0.003
Farmer	606(7.33)	12(7.64)			579(7.04)	11(5.42)			547(6.91)	31(6.08)		
Retired	3662(44.27)	83(52.87)			3681(44.75)	106(52.22)			3548(44.80)	274(53.73)		
Civil servant	690(8.34)	14(8.92)			692(8.41)	16(7.88)			672(8.49)	42(8.24)		
Professional/technical	1820(22.00)	24(15.29)			1783(21.68)	35(17.24)			1730(21.85)	84(16.47)		
Self-employed	793(9.59)	11(7.01)			769(9.35)	17(8.37)			760(9.60)	42(8.24)		
Freelancer	198(2.39)	4(2.55)			217(2.64)	6(2.96)			176(2.22)	15(2.94)		
Other	503(6.08)	9(5.73)			505(6.14)	12(5.91)			486(6.14)	22(4.31)		
Coronary artery disease, n (%)			χ²=0.11	0.737			χ²=0.06	0.81			χ²=5.21	0.022
No	6053(73.17)	113(71.97)			6019(73.17)	147(72.41)			5816(73.44)	351(68.82)		
Yes	2219(26.83)	44(28.03)			2207(26.83)	56(27.59)			2103(26.56)	159(31.18)		
Hypertensive heart disease, n (%)			χ²=0.67	0.413			χ²=0.10	0.747			χ²=0.11	0.739
No	8194(99.06)	154(98.09)			8144(99.00)	200(98.52)			7838(98.98)	504(98.82)		
Yes	78(0.94)	3(1.91)			82(1.00)	3(1.48)			81(1.02)	6(1.18)		
Myocardial infarction, n (%)			χ²=2.54	0.111			χ²=4.28	0.038			χ²=14.46	<0.001
No	8193(99.04)	153(97.45)			8157(99.16)	198(97.54)			7843(99.04)	496(97.25)		
Yes	79(0.96)	4(2.55)			69(0.84)	5(2.46)			76(0.96)	14(2.75)		
Lacunar infarction, n (%)			χ²=6.55	0.01			χ²=5.70	0.017			χ²=4.83	0.028
No	6994(84.55)	121(77.07)			6915(84.06)	158(77.83)			6673(84.27)	411(80.59)		
Yes	1278(15.45)	36(22.93)			1311(15.94)	45(22.17)			1246(15.73)	99(19.41)		
Cerebral hemorrhage, n (%)			–	1			–	1			χ²=0.78	0.377
No	8249(99.72)	157(100.00)			8202(99.71)	203(100.00)			7893(99.67)	510(100.00)		
Yes	23(0.28)	0(0.00)			24(0.29)	0(0.00)			26(0.33)	0(0.00)		
Peripheral artery disease, n (%)			χ²=0.14	0.705			χ²=0.00	1			χ²=3.10	0.078
No	8164(98.69)	156(99.36)			8127(98.80)	201(99.01)			7818(98.72)	508(99.61)		
Yes	108(1.31)	1(0.64)			99(1.20)	2(0.99)			101(1.28)	2(0.39)		
Diabetic nephropathy, n (%)			χ²=0.22	0.637			χ²=0.90	0.343			χ²=0.85	0.356
No	7369(89.08)	138(87.90)			7368(89.57)	186(91.63)			7090(89.53)	450(88.24)		
Yes	903(10.92)	19(12.10)			858(10.43)	17(8.37)			829(10.47)	60(11.76)		
CKD stage 3–5, n (%)			χ²=2.15	0.142			χ²=2.40	0.121			χ²=4.94	0.026
No	8040(97.20)	149(94.90)			8008(97.35)	194(95.57)			7709(97.35)	488(95.69)		
Yes	232(2.80)	8(5.10)			218(2.65)	9(4.43)			210(2.65)	22(4.31)		
Uremic stage of renal failure, n (%)			χ²=2.70	0.1			χ²=10.50	0.001			χ²=2.31	0.128
No	8195(99.07)	153(97.45)			8168(99.29)	197(97.04)			7854(99.18)	502(98.43)		
Yes	77(0.93)	4(2.55)			58(0.71)	6(2.96)			65(0.82)	8(1.57)		
Diabetic retinopathy, n (%)			χ²=2.65	0.104			χ²=4.94	0.026			χ²=13.26	<0.001
No	5111(61.79)	87(55.41)			5089(61.86)	110(54.19)			4911(62.02)	275(53.92)		
Yes	3161(38.21)	70(44.59)			3137(38.14)	93(45.81)			3008(37.98)	235(46.08)		
Diabetic neuropathy, n (%)			–	1			–	1			–	1
No	8265(99.92)	157(100.00)			8218(99.90)	203(100.00)			7910(99.89)	510(100.00)		
Yes	7(0.08)	0(0.00)			8(0.10)	0(0.00)			9(0.11)	0(0.00)		
Hypertension, n (%)			χ²=0.08	0.774			χ²=0.92	0.337			χ²=2.60	0.107
No	3625(43.82)	67(42.68)			3601(43.78)	82(40.39)			3488(44.05)	206(40.39)		
Yes	4647(56.18)	90(57.32)			4625(56.22)	121(59.61)			4431(55.95)	304(59.61)		
Dyslipidemia, n (%)			χ²=4.52	0.034			χ²=6.96	0.008			χ²=7.38	0.007
No	7158(86.53)	145(92.36)			7139(86.79)	189(93.10)			6875(86.82)	464(90.98)		
Yes	1114(13.47)	12(7.64)			1087(13.21)	14(6.90)			1044(13.18)	46(9.02)		
Pneumonia, n (%)			χ²=0.00	1			χ²=0.06	0.804			χ²=0.05	0.83
No	8199(99.12)	156(99.36)			8151(99.09)	202(99.51)			7841(99.02)	506(99.22)		
Yes	73(0.88)	1(0.64)			75(0.91)	1(0.49)			78(0.98)	4(0.78)		
COPD, n (%)			χ²=25.64	<0.001			χ²=6.46	0.011			χ²=4.63	0.031
No	8146(98.48)	146(92.99)			8105(98.53)	195(96.06)			7799(98.48)	496(97.25)		
Yes	126(1.52)	11(7.01)			121(1.47)	8(3.94)			120(1.52)	14(2.75)		
Sleep apnea syndrome, n (%)			–	0.483			χ²=0.19	0.664			χ²=0.00	1
No	8238(99.59)	156(99.36)			8184(99.49)	201(99.01)			7879(99.49)	507(99.41)		
Yes	34(0.41)	1(0.64)			42(0.51)	2(0.99)			40(0.51)	3(0.59)		
Sleep disorder, n (%)			χ²=30.35	<0.001			χ²=27.37	<0.001			χ²=40.08	<0.001
No	8121(98.17)	144(91.72)			8070(98.10)	188(92.61)			7777(98.21)	480(94.12)		
Yes	151(1.83)	13(8.28)			156(1.90)	15(7.39)			142(1.79)	30(5.88)		
Depression, n (%)			χ²=0.00	1			χ²=0.01	0.942			χ²=0.19	0.663
No	8209(99.24)	156(99.36)			8161(99.21)	202(99.51)			7863(99.29)	505(99.02)		
Yes	63(0.76)	1(0.64)			65(0.79)	1(0.49)			56(0.71)	5(0.98)		
Anxiety, n (%)			χ²=0.00	0.958			χ²=0.33	0.569			χ²=0.57	0.449
No	8189(99.00)	156(99.36)			8129(98.82)	202(99.51)			7828(98.85)	506(99.22)		
Yes	83(1.00)	1(0.64)			97(1.18)	1(0.49)			91(1.15)	4(0.78)		
Osteoarthritis, n (%)			χ²=0.41	0.521			χ²=0.01	0.913			χ²=0.68	0.41
No	8208(99.23)	157(100.00)			8159(99.19)	202(99.51)			7853(99.17)	508(99.61)		
Yes	64(0.77)	0(0.00)			67(0.81)	1(0.49)			66(0.83)	2(0.39)		
Gout, n (%)			–	1			χ²=0.38	0.536			χ²=0.54	0.461
No	8225(99.43)	157(100.00)			8178(99.42)	203(100.00)			7876(99.46)	509(99.80)		
Yes	47(0.57)	0(0.00)			48(0.58)	0(0.00)			43(0.54)	1(0.20)		
Osteoporosis, n (%)			χ²=11.19	<0.001			χ²=12.30	<0.001			χ²=8.32	0.004
No	7830(94.66)	139(88.54)			7769(94.44)	180(88.67)			7491(94.60)	467(91.57)		
Yes	442(5.34)	18(11.46)			457(5.56)	23(11.33)			428(5.40)	43(8.43)		
Tumor, n (%)			χ²=2.11	0.146			χ²=4.73	0.03			χ²=2.58	0.108
No	7853(94.93)	145(92.36)			7785(94.64)	185(91.13)			7506(94.78)	475(93.14)		
Yes	419(5.07)	12(7.64)			441(5.36)	18(8.87)			413(5.22)	35(6.86)		

M: Median, Q1: 1st Quartile, Q3: 3rd Quartile.Z: Mann-Whitney test, χ2: Chi-square test, -: Fisher exact.

### 3.2 Feature selection

**Figure 2:**
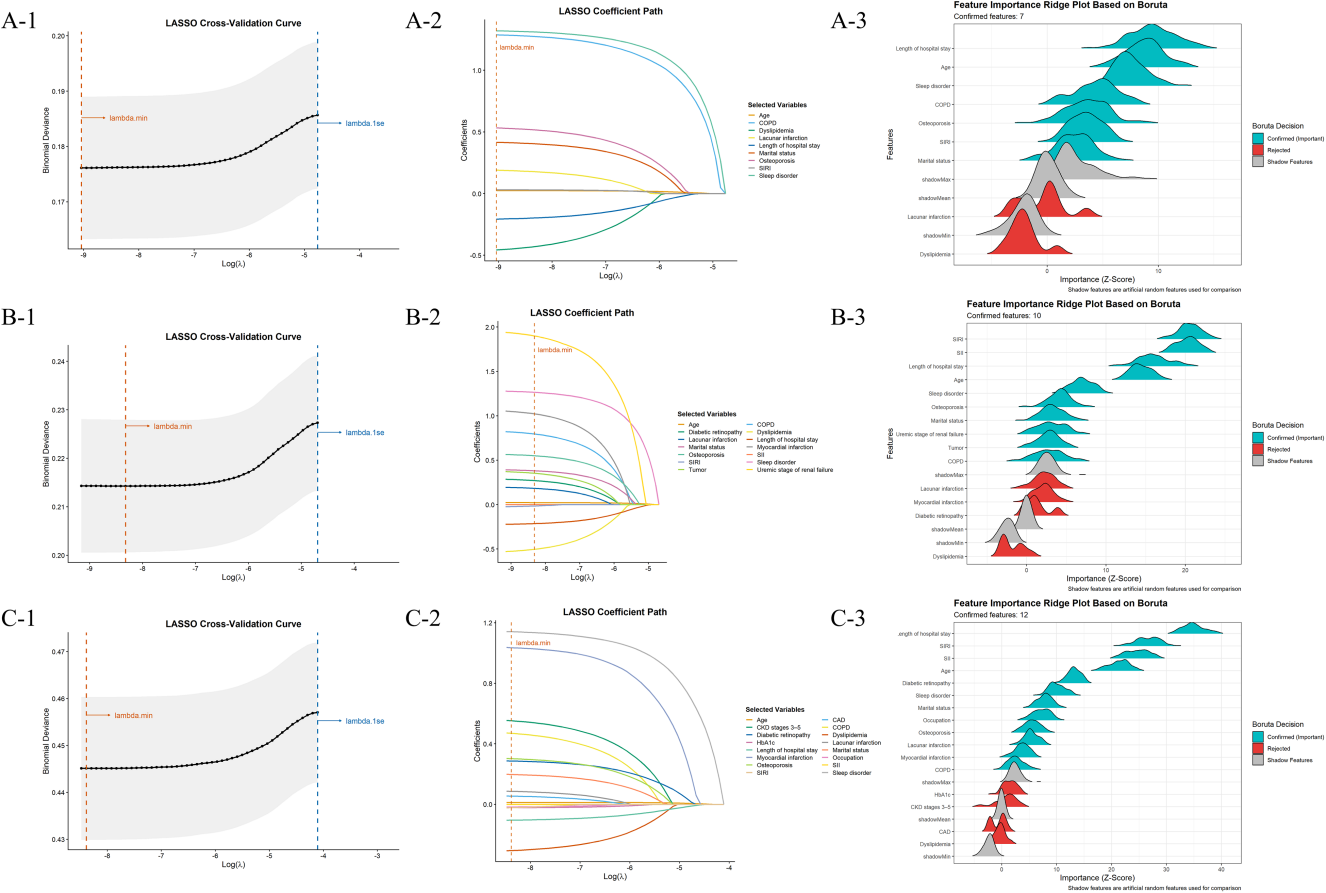
Variable selection using LASSO regression and Boruta algorithm. **(A)** 30-day readmission; **(B)** 60-day readmission; **(C)** 365-day readmission. Panels **(A-1, B-1, C-1)** show the 10-fold cross-validation plots of LASSO regression. Panels **(A-2, B-2, C-2)** present the coefficient path plots of LASSO regression. Panels **(A-3, B-3, C-3)** display the results of the Boruta algorithm.

Variable selection was performed using the training set. Variables that were statistically significant in univariate analysis were subsequently incorporated into LASSO regression and the Boruta algorithm. **Figure 2** illustrates the variable selection process for 30-day, 60-day, and 365-day readmission using LASSO regression (cross-validation plots and coefficient path plots) and the Boruta algorithm.

The final set of selected predictors for each time point was as follows:

30-day readmission: age, length of hospital stay, SIRI, marital status, COPD, sleep disorder, osteoporosis.

60-day readmission: age, length of hospital stay, SIRI, SII, marital status, uremic stage of renal failure, COPD, sleep disorder, osteoporosis, tumor.

365-day readmission: age, length of hospital stay, SIRI, SII, marital status, occupation, myocardial infarction, lacunar infarction, diabetic retinopathy, COPD, sleep disorder, osteoporosis.

Multicollinearity diagnostics showed no significant collinearity among the included predictors (all variance inflation factor VIF values <5).

### 3.3 Performance comparison of ML prediction models

**Figure 3:**
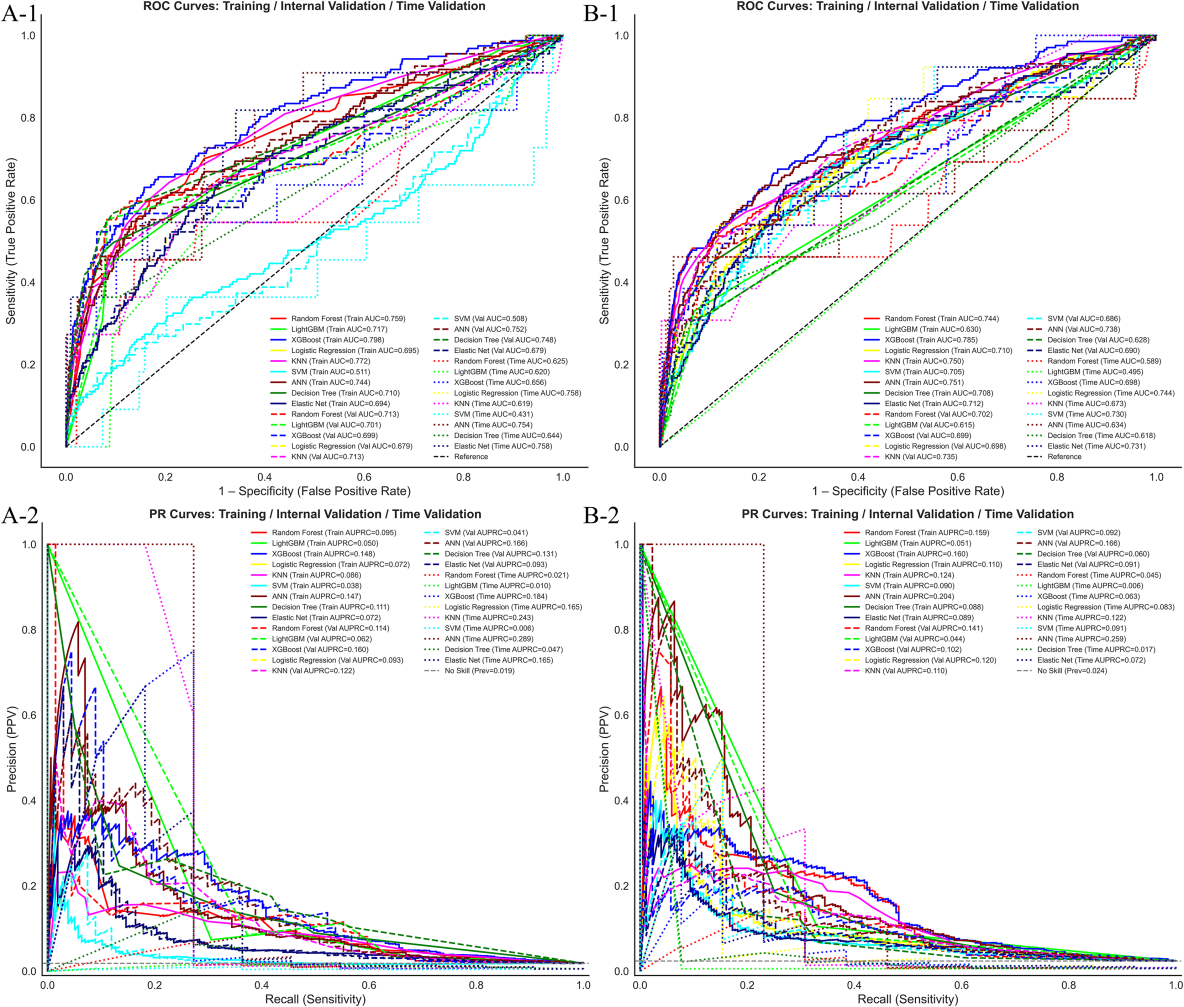
ROC and PR curves for 30-day and 60-day readmission predictions. **(A-1)** ROC curves for 30-day readmission; **(A-2)** PR curves for 30-day readmission; **(B-1)** ROC curves for 60-day readmission; **(B-2)** PR curves for 60-day readmission.

In this study, models for 30-day and 60-day readmission were evaluated on both an internal validation set (2018–2023 data, 30% random split) and an independent temporal validation set (2024 data). For 365-day readmission, due to incomplete follow-up for patients admitted in 2024, only internal validation results are reported. **Figure 3** presents the receiver operating characteristic (ROC) curves and precision-recall (PR) curves of the nine machine learning models for predicting 30-day and 60-day readmission in the training, internal validation, and temporal validation sets. The upper panels show ROC curves, and the lower panels show PR curves. The close alignment between the internal and temporal validation curves indicates consistent discriminative performance across the different validation time windows.

**Table 3** summarizes the performance of the nine models for predicting 30-day readmission. In the validation set, the ANN model achieved the highest AUROC (0.752, 95% CI: 0.681–0.816) and the highest AUPRC (0.166, 95% CI: 0.096–0.283). Decision tree (AUROC = 0.748) and random forest (AUROC = 0.713) also demonstrated good generalization performance. In the temporal validation set, ANN maintained robust performance (AUROC = 0.754, 95% CI: 0.576–0.908; AUPRC = 0.289, 95% CI: 0.009–0.587), with a sensitivity of 0.455 and good specificity (0.837).

**Table 3:** Performance of nine ML models for predicting 30-day readmission.

Variables	Model	AUROC	AUROC 95% CI lower	AUROC 95% CI upper	AUPRC	AUPRC 95% CI lower	AUPRC 95% CI upper	Brier score	Sensitivity	Specificity	F1 score
Training set	Random Forest	0.759	0.717	0.805	0.095	0.069	0.139	0.206	0.427	0.921	0.152
	LightGBM	0.717	0.675	0.762	0.050	0.038	0.065	0.018	0.440	0.918	0.152
	XGBoost	0.798	0.761	0.837	0.148	0.106	0.210	0.226	0.440	0.938	0.187
	Logistic Regression	0.695	0.649	0.739	0.072	0.048	0.114	0.216	0.497	0.788	0.079
	KNN	0.772	0.728	0.809	0.086	0.062	0.123	0.018	0.541	0.878	0.136
	SVM	0.511	0.458	0.564	0.038	0.024	0.067	0.018	0.140	0.938	0.064
	ANN	0.744	0.699	0.787	0.147	0.098	0.213	0.017	0.554	0.857	0.122
	Decision Tree	0.710	0.670	0.752	0.111	0.074	0.162	0.174	0.497	0.904	0.152
	Elastic Net	0.694	0.649	0.739	0.072	0.048	0.114	0.216	0.497	0.788	0.079
Validation set	Random Forest	0.713	0.624	0.789	0.114	0.066	0.187	0.206	0.552	0.920	0.191
	LightGBM	0.701	0.617	0.765	0.062	0.040	0.090	0.018	0.552	0.918	0.187
	XGBoost	0.699	0.607	0.777	0.160	0.092	0.268	0.226	0.522	0.938	0.217
	Logistic Regression	0.679	0.599	0.751	0.093	0.048	0.180	0.216	0.522	0.791	0.083
	KNN	0.713	0.622	0.783	0.122	0.068	0.215	0.018	0.493	0.877	0.123
	SVM	0.508	0.435	0.585	0.041	0.021	0.090	0.018	0.179	0.937	0.080
	ANN	0.752	0.681	0.816	0.166	0.096	0.283	0.017	0.537	0.860	0.120
	Decision Tree	0.748	0.680	0.808	0.131	0.078	0.215	0.172	0.567	0.906	0.174
	Elastic Net	0.679	0.599	0.751	0.093	0.048	0.180	0.216	0.522	0.790	0.083
Temporal validation set	Random Forest	0.625	0.414	0.827	0.021	0.006	0.071	0.209	0.273	0.909	0.031
	LightGBM	0.620	0.424	0.795	0.010	0.004	0.022	0.006	0.273	0.906	0.030
	XGBoost	0.656	0.428	0.860	0.184	0.006	0.503	0.226	0.273	0.939	0.044
	Logistic Regression	0.758	0.564	0.917	0.165	0.013	0.470	0.235	0.545	0.759	0.024
	KNN	0.619	0.387	0.821	0.243	0.008	0.503	0.005	0.273	0.896	0.027
	SVM	0.431	0.213	0.636	0.006	0.003	0.014	0.006	0.091	0.922	0.012
	ANN	0.754	0.576	0.908	0.289	0.009	0.587	0.006	0.455	0.837	0.029
	Decision Tree	0.644	0.499	0.810	0.047	0.005	0.164	0.171	0.364	0.933	0.054
	Elastic Net	0.758	0.564	0.917	0.165	0.013	0.470	0.235	0.545	0.758	0.024

AUROC, area under the receiver operating characteristic curve; AUPRC, area under the precision-recall curve; CI, confidence interval; ANN, artificial neural network; KNN, K-nearest neighbors; SVM, support vector machine. The Brier score measures calibration (0–1, lower is better). Sensitivity and specificity were calculated using the threshold that maximized the Youden index. The F1 score is the harmonic mean of precision and recall.

**Table 4** shows the performance for predicting 60-day readmission. In the validation set, ANN again achieved the highest AUROC (0.738, 95% CI: 0.679–0.797) and AUPRC (0.166, 95% CI: 0.099–0.254). In the temporal validation set, ANN had the highest AUPRC (0.259, 95% CI: 0.016–0.517), along with high specificity (0.871) and sensitivity (0.462).

**Table 4:** Performance of nine ML models for predicting 60-day readmission.

Variables	Model	AUROC	AUROC 95% CI lower	AUROC 95% CI upper	AUPRC	AUPRC 95% CI lower	AUPRC 95% CI upper	Brier score	Sensitivity	Specificity	F1 score
Training set	Random Forest	0.744	0.702	0.784	0.159	0.117	0.218	0.134	0.483	0.913	0.193
	LightGBM	0.630	0.600	0.664	0.051	0.039	0.065	0.024	0.330	0.930	0.159
	XGBoost	0.785	0.750	0.821	0.160	0.122	0.213	0.181	0.542	0.861	0.151
	Logistic Regression	0.710	0.668	0.749	0.110	0.076	0.157	0.023	0.700	0.580	0.075
	KNN	0.750	0.713	0.786	0.124	0.096	0.165	0.023	0.503	0.894	0.174
	SVM	0.705	0.669	0.744	0.090	0.064	0.129	0.023	0.567	0.751	0.097
	ANN	0.751	0.712	0.791	0.204	0.148	0.270	0.022	0.557	0.857	0.151
	Decision Tree	0.708	0.669	0.747	0.088	0.065	0.114	0.192	0.345	0.948	0.200
	Elastic Net	0.712	0.672	0.749	0.089	0.063	0.128	0.214	0.611	0.754	0.106
Validation set	Random Forest	0.702	0.638	0.766	0.141	0.087	0.231	0.138	0.494	0.900	0.178
	LightGBM	0.615	0.568	0.667	0.044	0.030	0.062	0.024	0.310	0.920	0.137
	XGBoost	0.699	0.636	0.767	0.102	0.068	0.167	0.185	0.529	0.854	0.142
	Logistic Regression	0.698	0.630	0.762	0.120	0.074	0.206	0.022	0.770	0.573	0.081
	KNN	0.735	0.674	0.795	0.110	0.071	0.163	0.023	0.506	0.881	0.160
	SVM	0.686	0.618	0.752	0.092	0.055	0.151	0.023	0.563	0.750	0.096
	ANN	0.738	0.679	0.797	0.166	0.099	0.254	0.024	0.517	0.848	0.135
	Decision Tree	0.628	0.565	0.690	0.060	0.039	0.091	0.196	0.299	0.946	0.172
	Elastic Net	0.690	0.623	0.753	0.091	0.059	0.154	0.217	0.575	0.751	0.099
Temporal validation set	Random Forest	0.589	0.378	0.803	0.045	0.010	0.147	0.129	0.385	0.898	0.045
	LightGBM	0.495	0.452	0.583	0.006	0.003	0.011	0.007	0.077	0.913	0.011
	XGBoost	0.698	0.534	0.853	0.063	0.011	0.215	0.184	0.385	0.846	0.031
	Logistic Regression	0.744	0.586	0.883	0.083	0.010	0.314	0.007	0.923	0.455	0.022
	KNN	0.673	0.526	0.832	0.122	0.016	0.389	0.006	0.308	0.907	0.040
	SVM	0.730	0.576	0.878	0.091	0.011	0.323	0.007	0.538	0.705	0.023
	ANN	0.634	0.431	0.839	0.259	0.016	0.517	0.008	0.462	0.871	0.043
	Decision Tree	0.618	0.449	0.789	0.017	0.006	0.050	0.183	0.231	0.966	0.072
	Elastic Net	0.731	0.572	0.876	0.072	0.010	0.279	0.238	0.538	0.701	0.023

AUROC, area under the receiver operating characteristic curve; AUPRC, area under the precision-recall curve; CI, confidence interval; ANN, artificial neural network; KNN, K-nearest neighbors; SVM, support vector machine. The Brier score measures calibration (0–1, lower is better). Sensitivity and specificity were calculated using the threshold that maximized the Youden index. The F1 score is the harmonic mean of precision and recall.

**Figure 4:**
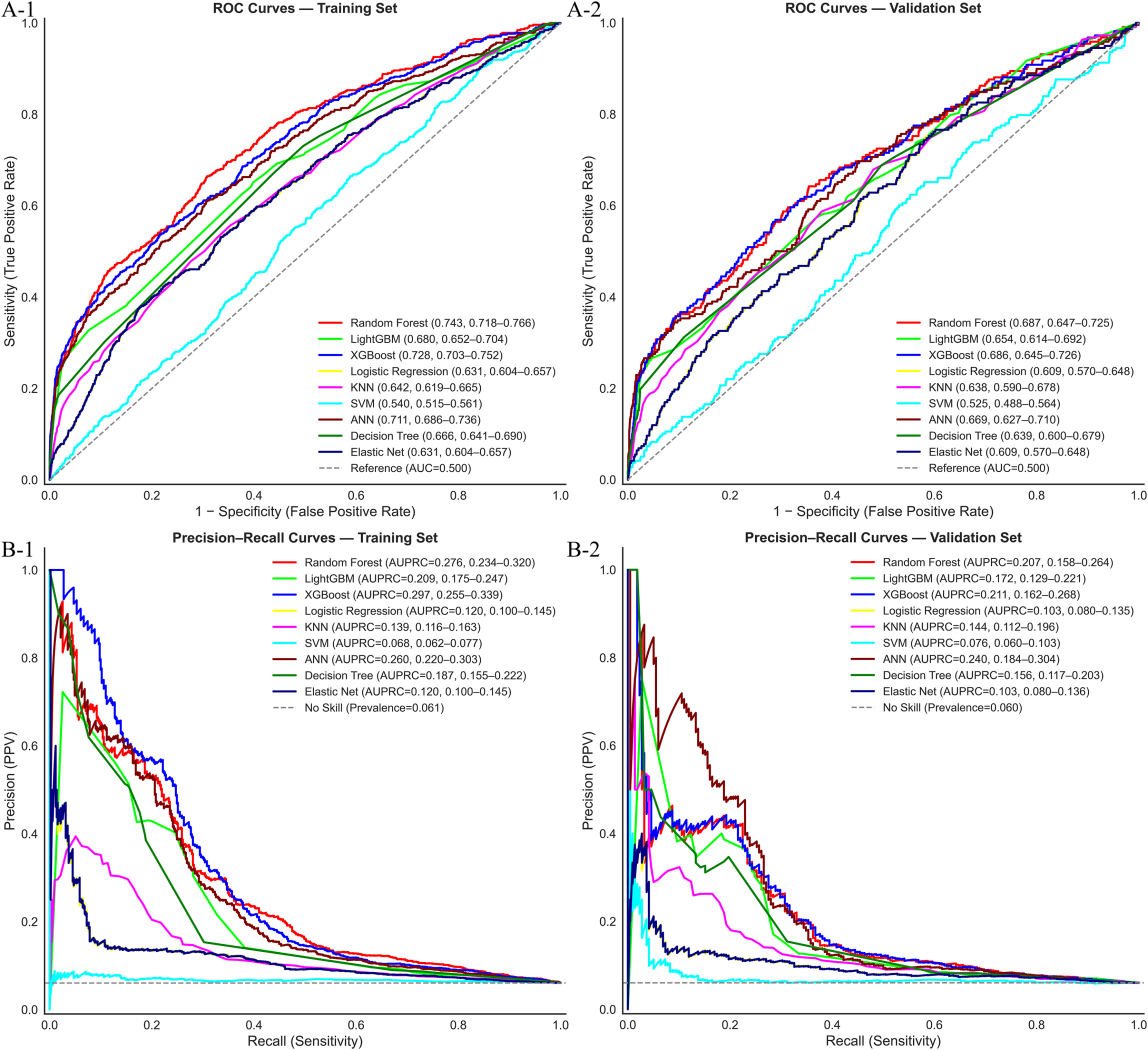
ROC and PR curves for 365-day readmission predictions. **(A-1)** ROC curves for 365-day readmission in the training set; **(A-2)** ROC curves for 365-day readmission in the internal validation set; **(B-1)** PR curves for 365-day readmission in the training set; **(B-2)** PR curves for 365-day readmission in the internal validation set.

**Figure 4** presents the ROC and PR curves for predicting 365-day readmission in the training and internal validation sets. **Table 5** summarizes the corresponding performance. In the validation set, random forest (RF) and XGBoost achieved comparable AUROC values (0.687 and 0.686, respectively). ANN had the highest AUPRC (0.240, 95% CI: 0.184–0.305) and a high specificity (0.910), with a good F1 score (0.250).

**Table 5:** Performance of nine ML models for predicting 365-day readmission.

Variables	Model	AUROC	AUROC 95% CI lower	AUROC 95% CI upper	AUPRC	AUPRC 95% CI lower	AUPRC 95% CI upper	Brier score	Sensitivity	Specificity	F1 score
Training set	Random Forest	0.743	0.718	0.766	0.276	0.234	0.320	0.200	0.708	0.634	0.191
	LightGBM	0.680	0.652	0.704	0.209	0.175	0.247	0.239	0.280	0.959	0.292
	XGBoost	0.728	0.703	0.752	0.297	0.255	0.339	0.202	0.604	0.707	0.196
	Logistic Regression	0.631	0.604	0.657	0.120	0.100	0.145	0.236	0.759	0.407	0.138
	KNN	0.642	0.619	0.665	0.139	0.116	0.163	0.056	0.578	0.614	0.153
	SVM	0.540	0.515	0.561	0.068	0.062	0.077	0.058	0.629	0.436	0.121
	ANN	0.711	0.686	0.736	0.260	0.220	0.303	0.051	0.382	0.904	0.266
	Decision Tree	0.666	0.641	0.690	0.187	0.155	0.222	0.212	0.302	0.893	0.203
	Elastic Net	0.631	0.604	0.657	0.120	0.100	0.145	0.236	0.767	0.388	0.136
Validation set	Random Forest	0.687	0.647	0.725	0.207	0.158	0.264	0.201	0.642	0.646	0.180
	LightGBM	0.654	0.614	0.692	0.172	0.129	0.221	0.239	0.266	0.954	0.268
	XGBoost	0.686	0.645	0.726	0.211	0.162	0.268	0.205	0.564	0.716	0.189
	Logistic Regression	0.609	0.570	0.648	0.103	0.080	0.135	0.235	0.752	0.416	0.139
	KNN	0.638	0.590	0.678	0.144	0.112	0.196	0.056	0.587	0.621	0.157
	SVM	0.525	0.488	0.564	0.076	0.060	0.103	0.057	0.624	0.448	0.122
	ANN	0.669	0.627	0.710	0.240	0.184	0.305	0.052	0.344	0.910	0.250
	Decision Tree	0.639	0.600	0.679	0.156	0.117	0.203	0.216	0.312	0.890	0.206
	Elastic Net	0.609	0.570	0.648	0.103	0.080	0.136	0.235	0.766	0.398	0.138

AUROC, area under the receiver operating characteristic curve; AUPRC, area under the precision-recall curve; CI, confidence interval; ANN, artificial neural network; KNN, K-nearest neighbors; SVM, support vector machine. The Brier score measures calibration (0–1, lower is better). Sensitivity and specificity were calculated using the threshold that maximized the Youden index. The F1 score is the harmonic mean of precision and recall.

**Figure 5:**
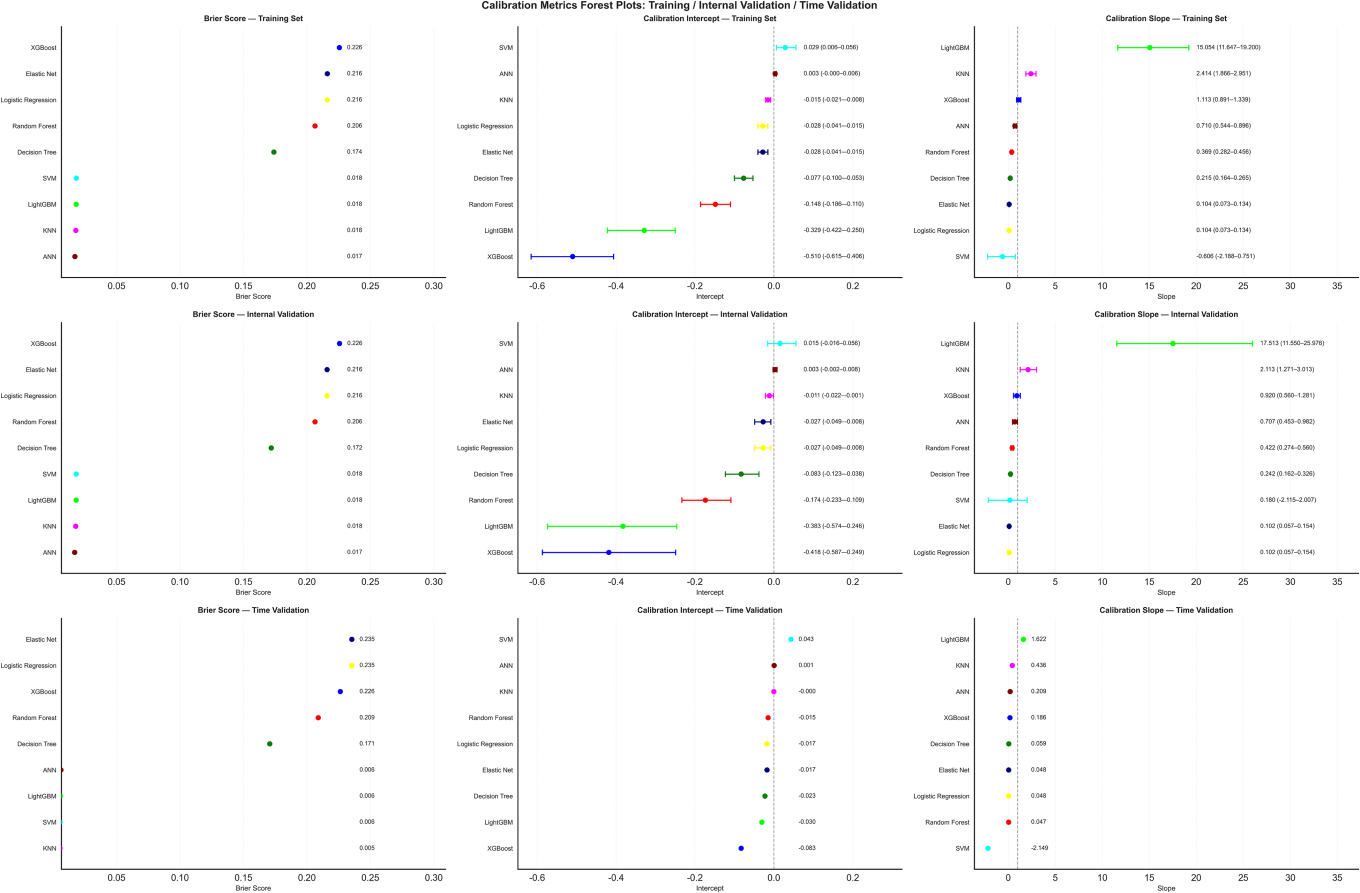
Calibration performance for 30-day readmission.

**Figure 6:**
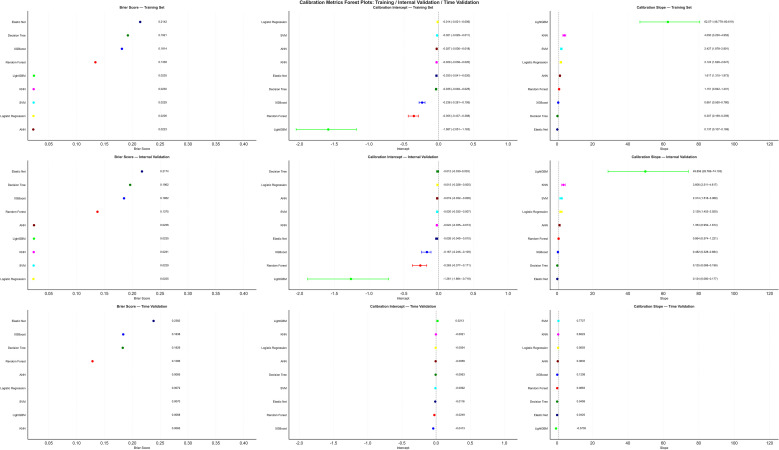
Calibration performance for 60-day readmission.

**Figures 5** and **6** compare the calibration performance (Brier score, calibration intercept, and calibration slope) of the nine models for 30-day and 60-day readmission, respectively. In the internal validation set, the calibration results of ANN are good, with slopes of 0.707 (30 days) and 1.354 (60 days). In the time validation set, ANN maintained a calibration intercept close to zero, but its calibration slopes decreased to 0.209 (30 days) and 0.383 (60 days), respectively.

**Figure 7:**
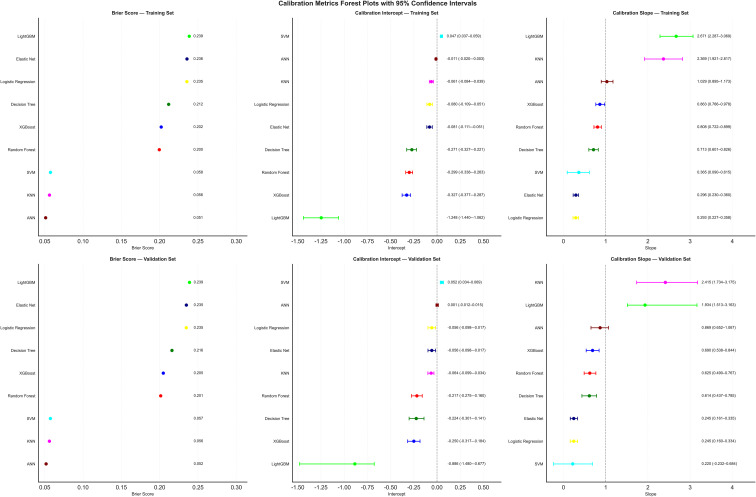
Calibration performance for 365-day readmission.

**Figure 7** shows calibration performance for 365-day readmission. ANN achieved the lowest Brier score (0.052) and the calibration intercept closest to zero, with a calibration slope of 0.869 — the closest to 1 among all models in the 365-day internal validation set.

**Figure 8:**
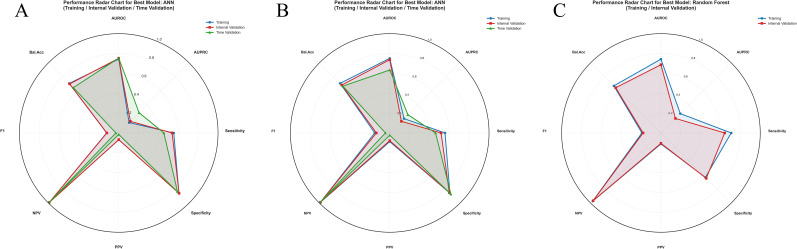
Radar plots of optimal models across time windows. **(A)** Radar plot of ANN performance for 30-day readmission in the training, internal validation, and temporal validation sets; **(B)** Radar plot of ANN performance for 60-day readmission; **(C)** Radar plot of random forest (RF) performance for 365-day readmission in the training and internal validation sets.

To visually compare the comprehensive performance of the optimal models across time windows, **Figure 8** presents radar plots. Based on AUROC performance, ANN was selected as the optimal model for 30-day and 60-day readmission, and random forest (RF) for 365-day readmission.

### 3.4 DeLong test for model comparison

**Figure 9:**
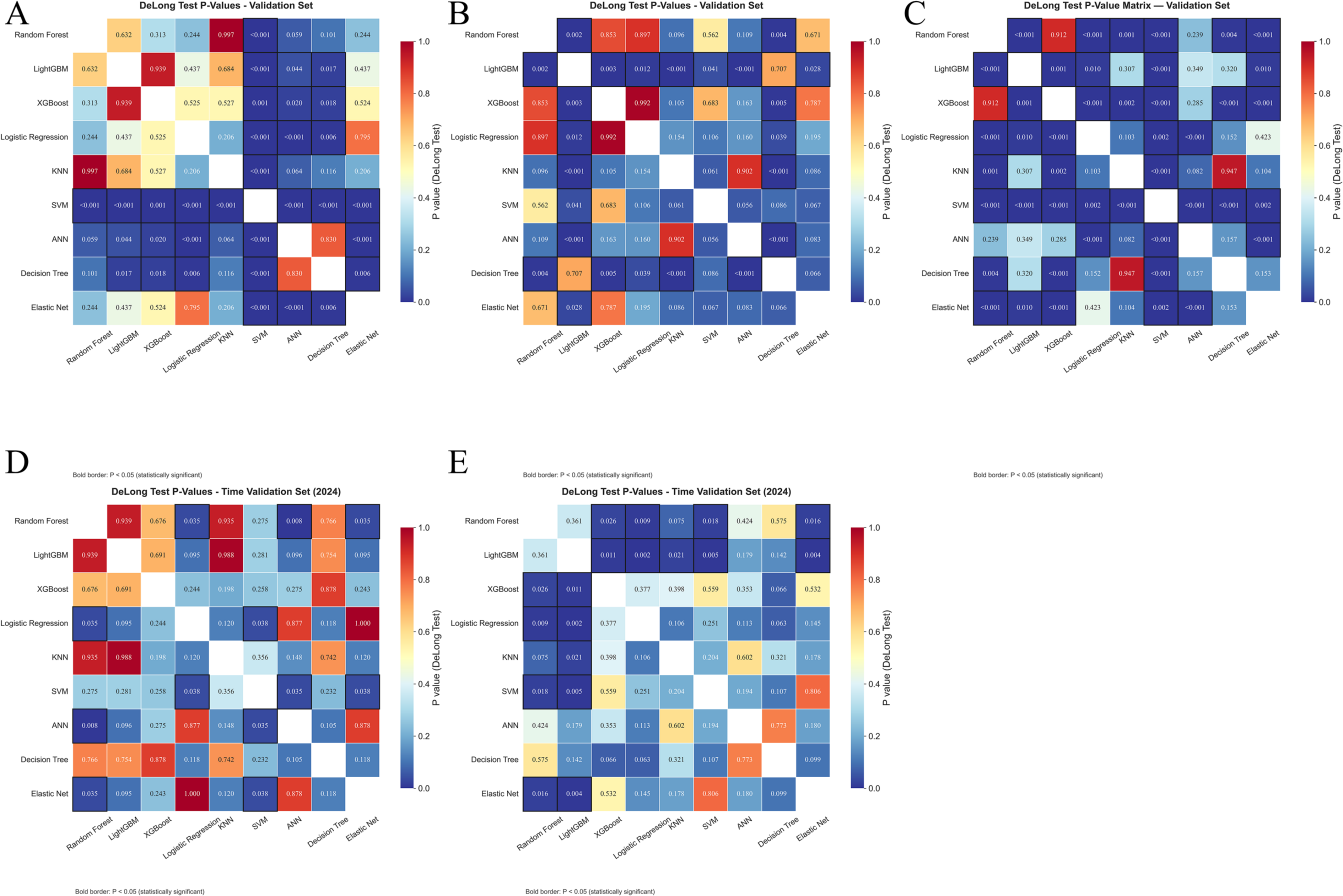
DeLong test heatmap. (A) 30‑day readmission (internal validation set); (B) 60‑day readmission (internal validation set); (C) 365‑day readmission (internal validation set); (D) 30‑day readmission (temporal validation set); (E) 60‑day readmission (temporal validation set).

DeLong tests were performed to compare the AUROC of the optimal model against the other eight models in the internal validation and temporal validation sets **Figure 9**.

30-day readmission: ANN significantly outperformed most models in the internal validation set (all *P* < 0.05), except for random forest, KNN, and decision tree. In the temporal validation set, ANN was superior only to random forest (*P* = 0.008) and SVM (*P* = 0.035).

60-day readmission: In the internal validation set, ANN significantly outperformed LightGBM and decision tree (both *P* < 0.001) but was not significantly different from the other models (including KNN, *P* = 0.902). In the temporal validation set, no significant differences were observed between ANN and any other model (all *P* > 0.05).

365-day readmission: In the internal validation set, random forest (RF) significantly outperformed LightGBM, logistic regression, KNN, SVM, decision tree, and elastic net (all *P* < 0.01) but was not significantly different from ANN (*P* = 0.239) or XGBoost (*P* = 0.912).

### 3.5 SHAP-based interpretability of the ANN model (30-day, 60-day) and RF model (365-day)

**Figure 10:**
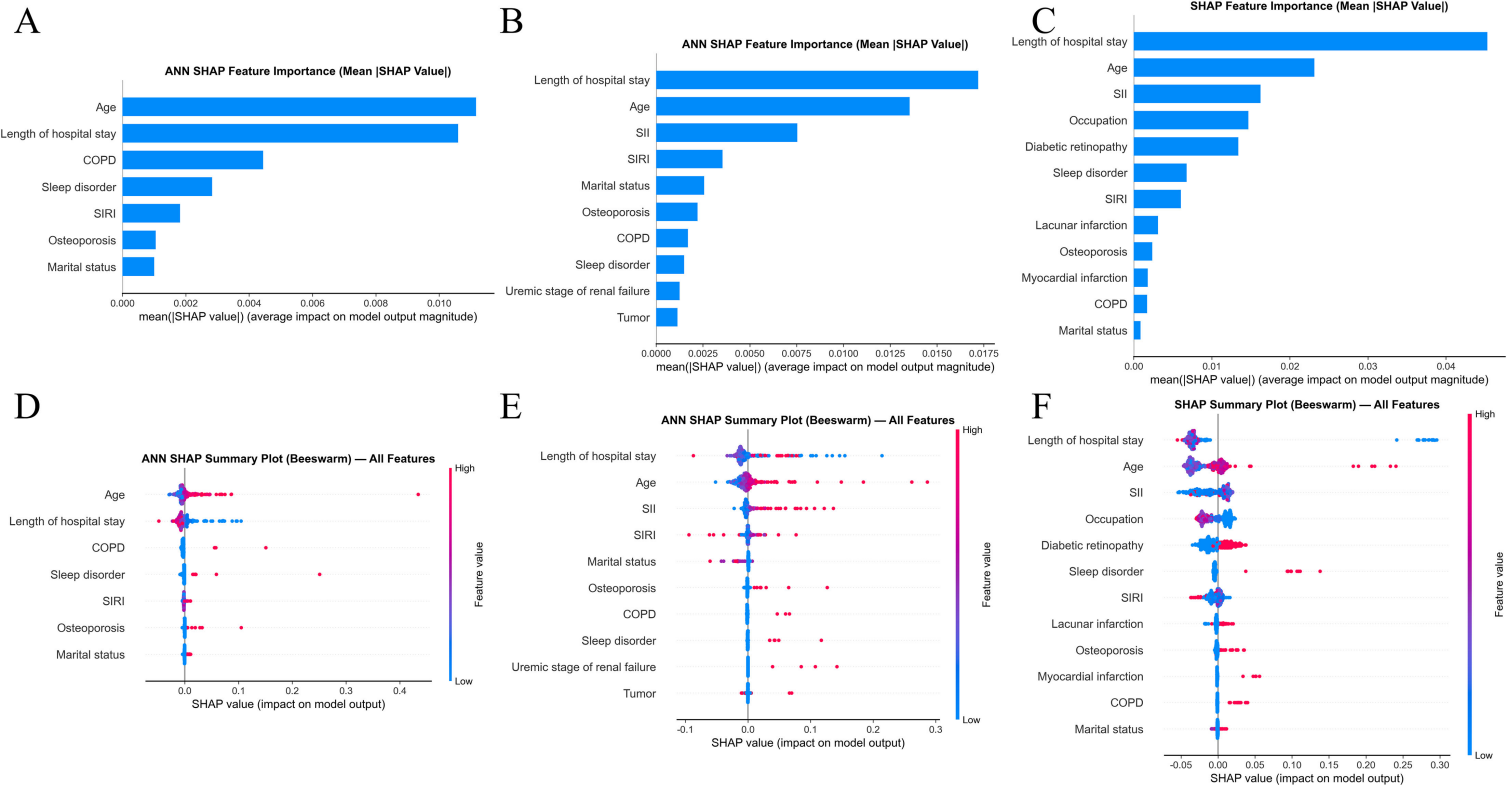
SHAP-based interpretability of ANN model (bar and beeswarm plots). **(A)** Bar plot for 30-day readmission; **(B)** Bar plot for 60-day readmission; **(C)** Bar plot for 365-day readmission; **(D)** Beeswarm plot for 30-day readmission; **(E)** Beeswarm plot for 60-day readmission; **(F)** Beeswarm plot for 365-day readmission.

To enhance model transparency and clinical interpretability, SHAP analysis was performed on the ANN model for the 30-day and 60-day readmission prediction tasks, and on the random forest (RF) model for the 365-day readmission task. **Figure 10** shows SHAP feature importance bar plots and beeswarm plots for each time window. **Table 6** summarizes the corresponding mean |SHAP| values.

**Table 6:** Comparison of SHAP-based feature importance (mean |SHAP|) for 30-day, 60-day, and 365-day readmission predictions.

Feature	30-day (ANN)	60-day (ANN)	365-day (RF)
Length of hospital stay	0.0106	0.0172	0.0453
Age	0.0112	0.0135	0.0231
SII	—	0.0075	0.0162
Occupation	—	—	0.0147
Diabetic retinopathy	—	—	0.0134
Sleep disorder	0.0028	0.0015	0.0068
SIRI	0.0018	0.0035	0.0061
Lacunar infarction	—	—	0.0031
Osteoporosis	0.0010	0.0022	0.0024
Myocardial infarction	—	—	0.0018
COPD	0.0044	0.0017	0.0017
Marital status	0.0010	0.0026	0.0009
Uremic stage of renal failure	—	0.0012	—
Tumor	—	0.0011	—

“—” indicates the feature was not selected as a predictor for that time window.

#### 3.5.1 SHAP analysis for 30-day readmission (ANN)

For 30-day readmission, the most important predictor was age (mean |SHAP| = 0.0112), followed by length of hospital stay (0.0106), COPD (0.0044), sleep disorder (0.0028), SIRI (0.0018), osteoporosis (0.0010), and marital status (0.0010). The beeswarm plot showed that age and length of stay had wide SHAP value distributions, with longer length of stay positively contributing to readmission risk.

#### 3.5.2 SHAP analysis for 60-day readmission (ANN)

For 60-day readmission, length of hospital stay became the most important feature (mean |SHAP| = 0.0172), followed by age (0.0135), SII (0.0075), SIRI (0.0035), marital status (0.0026), osteoporosis (0.0022), COPD (0.0017), sleep disorder (0.0015), uremic stage of renal failure (0.0012), and tumor (0.0011). Compared with the 30-day window, systemic inflammation markers (SII and SIRI) showed substantially increased importance.

#### 3.5.3 SHAP analysis for 365-Day readmission (random forest)

For 365-day readmission, based on the optimal random forest (RF) model, the most important predictor was length of hospital stay (mean |SHAP| = 0.0453), followed by age (0.0231), SII (0.0162), occupation (0.0147), diabetic retinopathy (0.0134), sleep disorder (0.0068), SIRI (0.0061), lacunar infarction (0.0031), osteoporosis (0.0024), myocardial infarction (0.0018), COPD (0.0017), and marital status (0.0009). The beeswarm plot indicated that higher values of length of stay, age, SII, and diabetic retinopathy consistently increased readmission risk.

#### 3.5.4 Dynamic trends in feature importance across time windows

Across the three time windows (**Table 6**), the following dynamic trends were observed:

Length of hospital stay ranked among the top predictors in all windows, with mean |SHAP| increasing progressively from 30 days (0.0106) to 60 days (0.0172) to 365 days (0.0453).

Age ranked first in the 30-day window and remained highly important across all windows, though its relative contribution decreased at 365 days (0.0231 vs. 0.0112 at 30 days in absolute terms, but lower rank).

Systemic inflammation markers (SII, SIRI) had low contributions in the 30-day window but became substantially more important in the 60-day and 365-day windows. SII emerged as the third most important feature at 365 days.

Diabetes complications (diabetic retinopathy, lacunar infarction, myocardial infarction) appeared prominently only in the 365-day window, whereas the 30-day and 60-day windows were dominated by comorbidities (COPD, osteoporosis, sleep disorders).

## 4 Discussion

This study systematically compared the performance of nine machine learning models in predicting 30-day, 60-day, and 365-day readmission in T2DM patients and revealed the dynamic evolution of risk factors across different time windows within the same large-sample clinical cohort. It should be noted that all readmission events analyzed in this study had T2DM as the primary diagnosis. The results showed that the artificial neural network (ANN) performed best in short-term (30-day) and medium-term (60-day) predictions, whereas random forest (RF) was slightly superior to the other models in long-term (365-day) prediction. In terms of calibration, ANN achieved near-ideal calibration intercepts across all windows. Its calibration slopes were 0.707 (30-day internal), 1.354 (60-day internal), and 0.869 (365-day internal). However, in the temporal validation sets, ANN’s calibration slopes decreased to 0.209 (30-day) and 0.383 (60-day), indicating that calibration may degrade when applied to future data — a common challenge in predictive modeling. SHAP analysis for the systematically demonstrated a dynamic evolution of risk factors from “acute vulnerability” to “inflammatory burden” and then to “chronic complication dominance.” These findings provide data support and a tool-based foundation for transitioning from static risk identification to dynamic risk monitoring in clinical practice.

### 4.1 Model selection across different time windows

This study found that ANN performed best for 30-day and 60-day readmission predictions, whereas RF was slightly superior for 365-day prediction. This difference is not coincidental but rather reflects fundamental differences in data structure across time windows.

Short-term readmission events are often driven by acute, nonlinear, and highly interactive factors. Infections can rapidly exacerbate metabolic disturbances; glycemic fluctuations aggravate systemic inflammatory responses through oxidative stress and endothelial dysfunction; prolonged hospital stay reflects the complexity of the initial illness or complications during medical care; and declining adherence to glucose management may stem from insufficient patient understanding or difficulty executing discharge self-management plans (24–27). These factors do not act independently but have complex interactions. Faced with this high-dimensional, nonlinear, strongly interactive data structure, ANN, with its multilayer nonlinear transformation capabilities and sensitivity to feature interactions, is better suited to capturing such complex patterns (28). Sangi et al. (29) showed that ANN can effectively capture interactions among covariates and outperform traditional regression models in predicting diabetes complications.

In contrast, long-term readmission risk depends more on stably accumulated chronic characteristics. Diabetes complications (retinopathy, myocardial infarction, lacunar infarction) require years or even decades of hyperglycemic exposure to develop; social support structures and occupational status reflect patients’ long-term living environment and resource availability. As an ensemble learning algorithm, RF is robust to outliers and noise through the voting mechanism of multiple decision trees; its built-in missing value handling capability (e.g., using surrogate variables for splitting) allows direct modeling without complex imputation strategies; moreover, RF can achieve stable performance in imbalanced data by adjusting class weights or decision thresholds (30). This suggests that model selection should be adapted to the time window of the prediction target. In future clinical deployment, a “time-window-specific model pool” could be constructed to achieve dynamic risk warning.

### 4.2 Time gradient of predictability

This study observed significant differences in model prediction performance across different time windows. Prediction of short-term (30-day) readmission was more difficult. This observation aligns with findings from previous readmission prediction studies that the difficulty may stem from the randomness and transient nature of its triggering factors.

Readmission within 30 days after discharge is often the result of multiple acute factors acting together. Sudden infection can cause a sharp rise in blood glucose within days; adjustments to the discharge medication regimen may lead to drug-induced hyperglycemia or hypoglycemia due to insufficient patient understanding or execution errors; and psychosocial stress (anxiety about disease prognosis, lack of family support) may affect patients’ self-management behaviors. These factors are highly context-dependent and time-sensitive, making them difficult to fully capture with static clinical variables at admission. Therefore, predicting 30-day readmission essentially faces the challenge of “incomplete information.”

In contrast, long-term (365-day) readmission more strongly reflects the cumulative effects of a chronic disease course. The development of microvascular (retinopathy, nephropathy) and macrovascular (coronary artery disease, cerebrovascular disease) complications is an inevitable consequence of long-term hyperglycemic exposure and has a clear dose–response relationship. Hyperglycemia gradually impairs vascular endothelial function through multiple pathways, including the formation of advanced glycation end-products (AGEs), oxidative stress, activation of protein kinase C (PKC), and the polyol pathway, eventually leading to clinically recognizable complications. This process is known as the “metabolic memory” effect – even if glycemic control improves later, the damage caused by earlier hyperglycemia continues to progress (31, 32). Anjana et al. (33) followed 3,581 T2DM patients for up to 9 years and found that the glycemic burden in the irregular follow-up group was twice that of the regular follow-up group, and the risks of developing retinopathy and nephropathy increased by 98% and 111%, respectively.

### 4.3 Temporal evolution of risk factors

SHAP analysis revealed significant dynamic evolution of risk factors across the three time windows, which is one of the core innovative findings of this study. In the short-term (30-day) window, age was the most important predictor, consistent with extensive literature reporting that elderly patients are prone to adverse events after discharge (34–36). Raval et al. (37) analyzed 202,496 elderly Medicare beneficiaries and found that 30-day readmission rates among elderly T2DM patients ranged from 11% to 23%. Age was followed by length of hospital stay, COPD, and sleep disorders. This indicates that short-term readmission primarily reflects the patient’s “acute vulnerability,” where older age, underlying pulmonary disease, and complex hospitalization collectively constitute a high-risk background for early post-discharge adverse events. Chaugule et al. (38) also found in diabetic or stress-hyperglycemic patients hospitalized for COVID-19 that older age, lower eGFR, comorbidity burden, ICU admission, and longer initial hospital stay were significantly associated with readmission risk. This suggests that the focus of intervention for short-term readmission should be on early high-risk identification after discharge and enhanced transitional care.

In the medium-term (60-day) window, length of hospital stay replaced age as the most important predictor. The persistently high importance of length of stay suggests that “process factors” experienced during hospitalization – such as intensity of medical intervention, management of complications, and difficulty of glycemic control – continue to influence the patient’s recovery trajectory during the subacute post-discharge period. Concurrently, the importance of systemic inflammation markers (SII, SIRI) increased significantly, suggesting that the core mechanism of medium-term readmission risk is transitioning from “baseline vulnerability” to “inflammatory burden” and “hospitalization process complexity.” This is consistent with Zhang et al. (39), who reported that SIRI is a key indicator for assessing inflammatory burden and prognosis in critically ill diabetic patients. The marked increase in marital status (social support) also indicates that social support during the subacute recovery period plays a critical role in preventing readmission.

In the long-term (365-day) window, length of stay remained the most important feature. More importantly, diabetes-specific chronic complications – diabetic retinopathy, lacunar infarction, and myocardial infarction – became the dominant predictors for the first time. This change has profound pathophysiological significance: long-term readmission is no longer the result of a single acute event but rather a systemic manifestation of cumulative microvascular and macrovascular damage caused by chronic hyperglycemia. This view is highly consistent with mainstream understanding in the field of international diabetes complications research. The MSD manual systematically describes the pathophysiological process by which hyperglycemia leads to microvascular and macrovascular lesions through mechanisms including AGEs, oxidative stress, and PKC activation. Fabris et al. (40) in Italy and Anjana et al. (33) in India have demonstrated the temporal accumulation and progression of vascular damage from longitudinal data. The SHAP value of diabetic retinopathy was as high as 0.0134, nearly equal to that of length of stay, indicating that it is not only an ocular lesion but also a “window” into systemic disease progression.

### 4.4 Clinical value of SHAP

Traditional predictive models often only provide a binary judgment of “high risk or not.” In this study, the SHAP beeswarm plot showed that age had a wide distribution and consistent direction (positive contribution) in the short-term window, indicating that age is a baseline risk factor for short-term readmission in almost all patients. In the long-term window, high SHAP values for diabetic retinopathy appeared only in a subset of patients, suggesting it is a highly specific key marker suitable for stratified management. This “individualized attribution” capability enables clinicians to develop intervention strategies based on risk mechanisms: for patients dominated by inflammation, anti-inflammatory or metabolic control should be strengthened; for those dominated by social support, home follow-up or community care should be enhanced.

### 4.5 Limitations

This study has several limitations. First, the single-center retrospective cohort design may limit generalizability; future multicenter external validation is needed. Second, some potential variables (e.g., adherence to glucose-lowering medications, health literacy, dietary patterns, and glycemic variability metrics) were not included in the analysis, which may affect the completeness of the models. Third, the definition of readmission was limited to events with T2DM as the primary diagnosis and re-admission to the same hospital, which may underestimate the actual readmission rate. Future research should further integrate multimodal data and combine them with a dynamic learning framework to achieve real-time risk warning and individualized intervention.

## 5 Conclusion

This study developed interpretable machine learning models to predict 30-day, 60-day and 365-day readmission in T2DM patients. ANN performed best for short- and medium-term predictions, while random forest was optimal for long-term prediction. SHAP analysis revealed a dynamic evolution of risk factors: age and acute vulnerability dominated the 30-day window; inflammatory burden emerged at 60 days; and diabetes-specific chronic complications became predominant at 365 days. These findings support a shift from static risk identification to dynamic, time-window-specific risk monitoring in clinical practice.

## Data Availability

The original contributions presented in the study are included in the article/supplementary material. Further inquiries can be directed to the corresponding author.
